# Evaluation of the impact of a targeted hygiene intervention on public hygiene understanding and behaviour

**DOI:** 10.3389/fpubh.2026.1807568

**Published:** 2026-05-07

**Authors:** Sally F. Bloomfield, Lisa M. Ackerley

**Affiliations:** 1 International Scientific Forum on Home Hygiene, Montacute, Somerset, United Kingdom; 2 Royal Society for Public Health, London, United Kingdom

**Keywords:** behaviour change, cleanliness, COM-B, hygiene resilience, intervention, risk management, targeted hygiene

## Abstract

**Introduction:**

Health agencies worldwide have pledged to improve public resilience against epidemics, a future pandemic and antimicrobial resistance. This study sets out to quantify whether adopting a risk management approach (Targeted Hygiene) and communicating it through a storytelling video can improve hygiene comprehension and intention to adopt effective, resilient behaviours.

**Methods:**

An online trial was implemented with 2,539 adults. The Intervention Group (IG) watched a custom-designed Targeted Hygiene video, whilst the Control Group (CG) were shown a health video. Both were questioned about hygiene comprehension and behavioural intentions.

**Results:**

Questioning showed the IG were significantly more knowledgeable about how hygiene prevents infection transmission. However, faced with specific risk scenarios (handling raw foods, or someone who is infected) both groups could identify “key hygiene habits,” but neither was able to differentiate vital actions, from actions that would have little benefit in that scenario. In both groups, particularly the CG, there was misunderstanding about the difference between hygiene and cleanliness.

**Conclusion:**

People understand the need for hygiene practices, and what these practices should be, but lack understanding of when and where to apply them. The findings suggest that animated visual materials, now increasingly accessible and affordable, have the potential to enhance hygiene comprehension and dispel public misperceptions about cleanliness and hygiene which may be acting as barriers to uptake of effective and resilient hygiene behaviour.

## 1 Introduction

In response to the COVID-19 pandemic, health agencies have pledged investment in building hygiene resilience, aimed at making the public better able to adapt to risk including threats of future epidemics or a further pandemic and antimicrobial resistance (AMR) (1–3).

A 2021 consensus review by the International Scientific Forum on Home Hygiene (IFH) (4) concluded that, to enable the public to practise effective hygiene, we must look at it from their viewpoint and how they practise it as part of daily life at home, in public spaces and in situations such as outbreaks, or where someone is at increased risk of infection.

Since 1997, the IFH has been developing an approach based on risk management as a framework for developing effective and resilient hygiene behaviour (5). This approach, known as Targeted Hygiene, recognizes that controlling infection is not about trying to eliminate hazards posed by pathogens, by generically “keeping hands and surfaces clean”, but by targeting hygiene habits specifically, when and where needed, to halt movement of harmful microbes – the chain of infection - thus reducing the risk of exposure and infection (4, 6, 7).

Within this framework, risk assessment in westernized homes identifies nine key moments in daily life (including using the toilet and disposing of faecal material, handling raw foods, eating with fingers, clearing and cleaning up after having a meal or drink, coughing, sneezing etc., touching surfaces frequently touched by others, handling and laundering of clothing and linens, caring for pets and other domestic animals, disposing of waste) when harmful microbes are most likely to be spread leading to exposure and subsequent infection (4, 5, 8). Targeting hygiene practices at these key moments tackles the riskiest times for spread of respiratory, gastrointestinal, skin, eye and other infections. Because it addresses all common hygiene-related infections, it not only reduces endemic levels of infection but can be adapted to meet changing needs. For example, in an infectious disease outbreak, combinations of hygiene moments for halting spread can be advised, mandated or relaxed as necessary.

COVID-19 showed the importance of public hygiene behaviours not only in homes, but also in shared environments like offices, schools, hospitality venues, and transport. Responsibility for hygiene must be shared by facility managers and users, with management providing both cleanliness and enabling good hygiene practice. Since professionals’ habits may be shaped by early experiences, this has significant implications across healthcare, food, and hospitality sectors (9–11).

This paper describes an intervention to estimate whether, and to what extent, it is possible to build intention to practise Targeted Hygiene, based on the key moments approach. In addition, the data were used to examine the impact of public misunderstandings and misbeliefs about hygiene and cleanliness on hygiene comprehension.

## 2 Methods

### 2.1 Development and implementation of the trial

A desk-based online trial was designed by UK Behavioural Insights Team (BIT)^1^ in collaboration with IFH, then implemented, and analysed by BIT. A 7-min video was created by IFH in collaboration with BIT to explain principles of Targeted Hygiene (TH video). Because hygiene involves halting movement of invisible harmful microbes, storytelling and visualisation (12, 13) was adopted as the basis for video development. The TH video (14) showed how, in real-life scenarios, a family uses the “key moments” approach to raw food handling, preventing spread of respiratory (RT) and gastrointestinal (GI) infections, demonstrating how Targeted Hygiene protects against infection by halting the *journey of the germ*.

The trial was executed from 30th July–6th August 2024 with a representative sample of 2,539 English adults (Table 1) randomised, respectively, to an Intervention Group (IG) that viewed the TH video or a Control Group (CG) that viewed a hygiene-unrelated health video. Trial participants were required to watch the whole video before answering the questions. The trial and data analysis was carried out by BIT using their PREDICTIV platform.^2^ It was completed by participants in their own time, and they were remunerated if they completed it. Median time for completion was 12 m 29 s.

**Table 1:** Sociodemographic characteristics of the study populations.

Population characteristics		% total	Number of participants	
			Control group	Intervention group
Gender	Women	55%	666	720
	Men	45%	538	597
	Other	1%	8	10
Age	18–24	11%	126	141
	25–34	18%	218	230
	35–44	17%	193	238
	45–54	21%	258	256
	55+	31%	386	412
Region	South and East	34%	404	460
	North	30%	356	394
	Midlands	21%	264	271
	London	15%	188	202
Ethnicity	Asian	8%	95	97
	Black	7%	98	92
	Mixed/other	3%	34	52
Income	<£40,000	56%	463	508
	£40,000 and over	44%	364	403

BIT, in collaboration with IFH developed 11 sets of questions (Qs) designed to assess participants’ hygiene comprehension, and hygiene behaviour intentions in risk scenarios. These were given to both groups. For questions about comprehension and Targeted Hygiene (Q1-7,9,10), they were asked to select one or more answers or images from a list, which they considered correct or appropriate to the scenario. For Q8, 11 and 12 about intention to practise hygiene in risk situations, a 4-point Likert scale was used where participants were asked whether they were “very likely, somewhat likely, somewhat unlikely, unlikely” to perform each practice. This was considered appropriate for assessing intention to practise hygiene because it enables participants to express nuanced attitudes, which is critical for understanding behavioural intention. By removing a neutral midpoint, the scale reduces ambivalence and encourages respondents to indicate a directional tendency, thereby improving the interpretability of results.

### 2.2 Analysis of data from the intervention trial

As required in any trial, the analytical approach, as follows, was agreed and fixed ahead of data collection. It was decided not to include Q3 to in the trial as it was considered too leading in nature. The remaining questions were grouped into three data sets covering hygiene comprehension (Q1,2,4,5,6), behavioural intention in food handling (Q7, Q8), and in GI and RT infection risk scenarios (Q9,10,11,12). In designing questions, IFH used published scientific data and accepted principles of risk management in each scenario to determine whether an answer was correct and appropriate, or an incorrect or inappropriate response in context of the question.

Because there were variable numbers of correct answers to each question, answers to all questions were weighted as 1 for a correct answer or 0 for an incorrect or inappropriate answer. For questions using the Likert scale, selecting a “correct” response of either “very” or “somewhat” likely, or “unlikely” as appropriate was scored as “1. All “incorrect or inappropriate” responses were scored as “0″. Composite scores were created by summing scores for the questions in each of the three data sets. This enabled estimation of the intervention impact (the Treatment Effect), whilst maintaining scientific robustness.

Quasibinomial regression analysis was chosen as the appropriate method for comparing composite scores for the CGs and IGs and determining the Treatment Effect, whilst controlling for age and gender. For each of the three data sets, composite scores for CG responses (sum of correct answers for each question expressed as a percentage of the sum of all possible correct answers to all questions in the set) served as the baseline level of comprehension or intent. Comprehension or intent of the IG was obtained by summing the baseline level and Treatment Effect. Data were analysed to determine whether the impact was statistically significant (p = <0.05).

### 2.3 The impact of misunderstanding and misbeliefs about hygiene and cleanliness

In addition to analysing composite scores for the three data sets as part of the trial, individual questions within data sets were examined to see whether the CG was more likely than the IG, to select incorrect or inappropriate responses to the questions. Data were analysed using Yates’ continuity-corrected chi-square test for all 2 × 2 comparisons to provide a conservative estimate of differences between CG and IG responses, reducing the risk of inflated significance that can occur with discrete categorical data. The phi coefficient was used to provide a simple effect-size measure for binary 2 × 2 associations.

## 3 Results

### 3.1 Analysis of the BIT trial

#### 3.1.1 Comprehension of the targeted approach to hygiene

Questions about comprehension of the *journey of the germ*, and Targeted Hygiene are shown in Table 2. The TH video clearly stated that the common sources of harmful microbes are people, contaminated foods and domestic animals. Q1 shows the IG had better comprehension of sources of harmful microbes, although awareness of person-to-person spread was relatively poor for both groups. Q2 showed the IG also had better comprehension of how harmful microbes spread, particularly in relation to domestic pets.

**Table 2:** Comprehension of how harmful microbes are spread and the concept of targeted hygiene practices.

Percentage of participants that selected each option	Control (CG) (*n* = 1,212)	Targeted hygiene (IG) (*n* = 1,327)	IG-CG (pp)	Chi^2^ (Yates)	*p*-value	Phi

Question 1: How are most common germs that cause infections introduced into the home? Select 3 answers						
Raw foods such as meat, fish, poultry, vegetables, fruit	70%	79%	+9.0	26.68	**<0.0001**	0.10
People	68%	69%	+0.4	0.033	0.85	0.004
Pets	25%	47%	+22.0	130.5	**<0.0001**	0.23
The toilet	50%	43%	−6.1	9.25	**0.0024**	0.06
From outdoors in the air, on our shoes and clothes etc.	46%	33%	−12.9	43.98	**<0.0001**	0.13
Dirty floors and surfaces	41%	29%	−12.4	42.34	**<0.0001**	0.13
Question 2: How are harmful germs most commonly spread in the home? Select 6 answers						
When we cough, sneeze, talk loudly, sing	84%	90%	5.7	18.21	**<0.0001**	0.08
When we handle and prepare raw food	83%	89%	6.1	19.17	**<0.0001**	0.08
When we eat with our fingers without washing our hands	83%	84%	1.6	1.03	0.30	0.02
When we touch surfaces other people regularly touch	82%	81%	−0.5	0.07	0.78	0.005
When we use the toilet	78%	80%	2.1	1.58	0.20	0.03
When we handle, care for, stroke our pets	49%	70%	20.9	113.7	**<0.0001**	0.21
When they breed on dirty surfaces and spread	77%	60%	−17.1	83.94	**<0.0001**	0.18
When things get dirty	43%	27%	−15.3	64.81	**<0.0001**	0.16
When things get untidy and messy	22%	18%	−3.5	4.55	**0.03**	0.04
Question 3: When should you practise hygiene to reduce the spread of harmful germs? 1 choice only						
At key moments when there is risk of harmful germs spreading	22%	34%	+12.66	49.42	**<0.0001**	**0.14**
As often as you can	64%	52%	−11.08	31.43	**<0.0001**	0.11
When somebody is ill	6%	6%	**−0.31**	**0.06**	**0.80**	**0.005**
At least once a week	6%	5%	**−1.42**	**2.31**	**0.13**	**0.03**
When things look dirty	3%	3%	**+0.15**	**0.01**	**0.91**	**0.002**
Question 4: As part of daily routine, which of the following are key moments when you should perform good hygiene practices? Select 3 answers						
After handling raw foods	85%	88%	+2.9	4.50	**0.03**	0.04
Disinfecting surfaces that are touched frequently	81%	84%	+2.6	2.80	0.09	0.03
Before handling ready to eat foods	78%	81%	+3.0	3.23	0.07	0.04
When surfaces do not look clean	35%	29%	−6.1	10.65	**0.001**	0.07
When floors are visibly dirty	20%	18%	−2.4	2.153	0.14	0.03
Question 5: Click on the 3 images where Priya should prioritise good hygiene during her day to prevent the spread of harmful germs						
After Priya used the toilet	94%	93%	−1.2	1.27	0.25	0.02
After touching and caring for pets	92%	91%	−0.3	0.04	0.83	0.004
When Priya is doing her laundry	45%	46%	+0.7	0.11	0.73	0.007
As Priya checks her mail	39%	38%	−0.9	0.16	0.68	0.008
When Priya is watering plants	25%	24%	−1.1	0.32	0.56	0.01
When Priya reads a book	5%	8%	+2.7	7.00	**0.008**	0.05
Question 6: What do you think is the best way to reduce the risk of harmful germs spreading in your home? Choose 1 answer						
Recognising and acting at key moments where cleaning or handwashing will reduce spread of harmful germs	**33%**	**45%**	**+12.28**	**39.46**	**0.0001**	**0.13**
Keeping home clean and tidy and washing our hands frequently	36%	31%	**−5.39**	**8.05**	**0.004**	**0.06**
Deep cleaning home with antibacterial products as often as possible & washing hands frequently	31%	24%	−6.89	15.18	**<0.0001**	**0.08**

Responses highlighted in grey are considered incorrect or inappropriate in this scenario. *p*-values in bold are significant.

The TH video explained that, to be effective, hygiene practices must be targeted at moments when there is risk of harmful microbes spreading. Qs 3,4,5,6 were designed to see how well participants understood this and could identify key moments for hygiene. Q3 shows 34% of the IG (compared with CG, 22%) identified “practising hygiene at key moments,” whereas 64% of the CG (compared with 52% of the IG) said hygiene should be practised “as often as you can.” This is in line with Q6 which showed 45% of the IG (compared with 33% of the CG) selected Targeted Hygiene as the best way to reduce risk of spread of harmful germs. Given a list, or set of images (Q4 and 5), both the IG and CG showed a good level of comprehension, identifying 6 of the 9 key hygiene moments, although only about half identified laundry.

Composite scores for data set 1 (Q1, 2, 4, 5, 6) were combined and analysed to estimate the impact of the intervention on hygiene comprehension. Quasibinomial regression analysis of the combined data showed overall comprehension for the CG, who did not see the TH video, was 70%. For the IG, it was estimated to be 6 percentage points higher (76%) and was statistically significant (*p* ≤ 0.01). Data for individual questions (Table 2) indicate that the impact was primarily on comprehension of the *journey of the germ* and need for Targeted Hygiene. For eight of the responses to Q1-4, there was a greater than 12% and up to 22% improvement in hygiene comprehension in the IG compared with the CG. Both groups showed a high level of knowledge of key hygiene moments, indicating that the intervention had little impact here.

#### 3.1.2 Comprehension and behavioural intentions in real- life scenarios

Participants were questioned about comprehension of the *journey of the germ* in real-life scenarios, and intention to practise targeted behaviour. Responses are shown in Tables 3–5.

**Table 3:** Understanding the journey of the germ and intention to practise hygiene in a food hygiene scenario.

Percentage of participants that selected each option	Control, CG (*n* = 1,212)	Targeted hygiene, TH (*n* = 1,327)	TH-CG (pp)	Chi^2^ (Yates)	*p*-value	Phi
Question 7: Abi takes pack of chicken, cuts it on chopping board, puts in saucepan, adds water from the tap. Once it’s done, where are you most likely to find harmful germs? Select 3 answers						
The cutting board and knife	95%	93%	−1.7	2.77	0.09	0.03
Abi’s hands	92%	91%	−1.8	2.28	0.13	0.03
The sink taps	75%	75%	0.5	0.05	0.82	0.004
The kitchen sink	22%	25%	2.4	1.94	0.16	0.03
The kitchen floor	9%	8%	−1.2	1.10	0.29	0.02
The fridge	7%	8%	1.8	2.53	0.11	0.03
Question 8: After chopping raw meat and vegetables, how likely you are to do each of the following to stop harmful germs spreading (% who said “somewhat” or “very likely” to do this)						
Wash your hands	99%	97%	−2.22	12.02	**0.0005**	0.069
Clean surfaces that meat or vegetables have been in contact with	98%	97%	−0.94	1.85	0.17	0.03
Clean any utensils which touched the food	98%	96%	−1.64	5.79	0.016	0.05
Wash or replace any dish cloths used to clean the area	88%	91%	+3.23	6.77	**0.009**	0.05
Clean all work surfaces in your kitchen	87%	85%	−2.12	2.18	0.14	0.05
Clean the kitchen floor	48%	47%	−1.02	0.22	0.64	0.009
Wash the clothes you were wearing	38%	40%	+1.35	0.43	0.51	0.01

Responses highlighted in grey are considered incorrect or inappropriate in this scenario. *p*-values in bold are significant.

**Table 4:** Understanding the *journey of the germ* in gastrointestinal and respiratory infection scenarios.

Percentage of participants that selected each option	Control, CG (*n* = 600)	Targeted hygiene IG (*n* = 678)	IG-CG (pp)	Chi^2^ (Yates)	*p*-value	Phi
Question 9: Imagine you live with Tom, who is sick with vomiting and diarrhoea. When should you, Tom and other people you live with wash your hands to protect against getting Tom’s stomach bug? Select 3 answers						
After using the toilet	80%	78%	−1.9	0.60	0.43	0.02
After handling Tom’s soiled clothing or bedding	73%	70%	−2.8	1.10	0.29	0.03
Before eating foods with your hands	54%	62%	+8.6	9.30	**0.002**	0.08
After coughing and sneezing into your hands	61%	60%	−1.1	0.12	0.72	0.01
After handling raw meat	25%	22%	−3.5	1.97	0.16	0.04
After caring for pets	8%	8%	0.8	0.15	0.69	0.01
Question 10: Imagine you live with Susan, who has cough, fever, and a runny nose. When should everyone in the home prioritise handwashing to prevent them getting her illness? Select 3 answers						
	(*n* = 612)	(*n* = 649)				
After coughing or sneezing into hands	91%	87%	−3.2	2.87	0.09	0.05
After touching surfaces frequently touched by other people	81%	80%	−0.6	0.04	0.84	0.006
Before eating food with my fingers	68%	72%	+4.3	2.57	0.10	0.04
After handling raw meat	36%	34%	−2.2	0.58	0.44	0.02
After caring for pets	16%	18%	+2.3	1.05	0.30	0.03
When they look dirty	9%	8%	−0.6	0.09	0.75	0.009

Responses highlighted in grey are considered incorrect or inappropriate.in this scenario. *p*-values in bold are significant.

**Table 5:** Intention to practise hygiene in gastrointestinal and respiratory infection scenarios.

Percentage of participants that said “somewhat” or “very likely” to do this			Statistical analysis			
Gastrointestinal infection						
Question 11 When someone in your home is infected, how likely are you to do each of the following (in addition to washing your hands)	Control, CG (*n* = 600)	Targeted hygiene, IG (*n* = 678)	IG-CG (pp)	Chi^2^ (Yates)	*p*-value	Phi
Clean surfaces commonly touched by everyone at home	95%	92%	−2.5	2.88	0.08	0.05
Disinfect toilet after use by sick person	93%	90%	−2.3	1.79	0.18	0.04
Wash/replace cloths immediately after cleaning surfaces in kitchen and toilet	90%	90%	−0.5	0.03	0.85	0.005
Open windows	89%	89%	+0.2	0.001	0.97	0.001
Wear masks	44%	46%	+1.6	0.25	0.61	0.01
Deep clean your home	69%	70%	+0.4	0.01	0.91	0.003
Wear gloves	62%	60%	−1.7	0.31	0.57	0.02
Respiratory infection						
Question 12 When someone in your home is infected how likely are you to do each of the following (in addition to washing your hands)	Control (*n* = 612)	Targeted hygiene (*n* = 649)	IG-CG (pp)	Chi^2^ (Yates)	*p*-value	Phi
Clean surfaces commonly touched by everyone at home	91%	91%	−0.4	0.02	0.86	0.005
Disinfect toilet after use by sick person	81%	82%	+1.0	0.13	0.71	0.01
Wash/replace cloths immediately after cleaning surfaces in kitchen and toilet	80%	85%	+5.2	5.43	**0.019**	0.07
Open windows	88%	89%	+1.1	0.30	0.58	0.02
Wear masks	41%	44%	+1.9	0.38	0.53	0.02
Deep clean your home	61%	58%	−2.6	0.74	0.38	0.02
Wear gloves	41%	44%	+2.9	0.95	0.32	0.03

Responses highlighted in grey are considered incorrect or inappropriate in this scenario. *p*-values in bold are significant.

##### 3.1.2.1 Food hygiene

Participants were asked to imagine they were watching someone preparing raw chicken. Q7 was designed to assess comprehension of how microbes spread in that scenario by asking “where are you most likely to find harmful germs.” Data in Table 3 indicate no significant difference between the CG and IG. Both showed high awareness of routes of spread of microbes from chicken to the chopping board and hands (92–95%) and relatively high awareness of potential for transfer via hands to sink taps (75%).

Asked to identify actions they would take to prevent spread of harmful microbes when handling raw meat and vegetables (Q8), both groups expressed high level of intent to carry out correct practices including handwashing [99% (CG) and 97% (IG)] and cleaning of food contact surfaces [98% (CG) and 97% (IG)] and cleaning cloths [88% (CG) and 91% (IG)].

Composite scores for all questions in data set 2 (Q7 and Q8) were combined and analysed to estimate the impact on comprehension of infection transmission routes and behavioural intent during food handling. Quasibinomial regression analysis of the combined data showed overall scores for the CG was 92, and 91% for the IG and there was no significant difference between the IG and CG.

##### 3.1.2.2 Gastrointestinal and respiratory illness

For the GI and RT illness scenarios (Q9-12), the CG and IG were further randomised into two sub-groups, ensuring responses for one scenario were not influenced by the other. CG and IG sub-groups were asked to imagine they were living with someone who either had an RT infection or someone who had a GI infection. Firstly, they were questioned about comprehension of how harmful microbes are spread in the scenario, by asking when they would wash their hands (Table 4). They were then asked about behavioural intentions to prevent spread of infection to other household members (Table 5). For Q11 and 12, CG and IG participants were given the same seven answer options for both scenarios.

For the GI infection subgroups, there was little difference between the CG and IG. Answers to Q9 (Table 4) showed high awareness of potential for transmission of microbes responsible for GI infections via the toilet [80% (CG) and 78% (IG)], soiled clothing [73% (CG) and 70% (IG)], indicating understanding of the *journey of the germ.* However, awareness that transmission can occur via soiled hands to ready to eat food was less, particularly for the CG [54% (CG) and 62% (IG)]. Asked about behavioural intentions (Table 5, Q11), both groups showed high awareness of the importance (in addition to handwashing) of disinfecting the toilet after use by a sick person, cleaning surfaces touched by others and decontaminating cleaning cloths; responses from both CG and IG were between 90 and 95%.

Table 4 shows responses to Q10 for RT infection sub-group. Both CG and IG showed a high level of awareness of routes of infection transmission by coughing and sneezing [91% (CG) and 87% (IG)] and touching surfaces frequently touched by others [81% (CG) and 80% (IG)], but only 68 and 72%, respectively, associated eating food with fingers with transmission. However, a third of both groups incorrectly thought handwashing after handling raw meat was also needed to reduce spread of RT infection. Asked about behavioural intentions (Table 5, Q12), both groups had high awareness of the importance (in addition to handwashing) of ventilation, disinfecting cleaning surfaces touched by others and washing or replacing cloths immediately after use to clean surfaces in the kitchen and toilet with responses from both groups between 80 and 91%. However, only 41 and 44%, respectively, said they were “somewhat or very likely to wear masks.”

Composite scores for data set 3 (Q9-Q12) were combined and analysed to estimate the impact on comprehension of infection transmission routes and behaviour intentions in GI and RT illness scenarios. Quasibinomial regression analysis of the combined data showed comprehension of infection transmission and behavioural intentions scores were 79% for both the CG and the IG and therefore there was no significant difference between the groups.

### 3.2 The impact of misunderstanding and misbeliefs about hygiene and cleanliness

#### 3.2.1 Intention to practise hygiene actions not appropriate to the risk scenario

Having noted that improved hygiene comprehension did not increase intention of either the CG or IG to practise Targeted Hygiene in raw food handling scenarios or when exposed to infection, data in Tables 3–5 were analysed further to determine whether the CG was more likely to choose incorrect or inappropriate responses. Both CG and IG expressed a level of intent to carry out inappropriate behaviours in risk scenarios. In all three scenarios, there was no significant difference between the CG and IG groups; the CG and IG were equally likely to select inappropriate responses that would have little or no impact in that scenario:

- In the food handling scenario (Table 3, Q8) both CG and IG groups showed intention to practise hygiene on all kitchen surfaces, (87 and 85%), not just those associated with spread of pathogens, indicating a belief that “general kitchen cleanliness” prevents spread of microbes from raw foods. This included cleaning the kitchen floor (48 and 47%) and washing clothes they were wearing” (38 and 40%).
- In the GI scenario (Table 5, Q11) both CG and IG groups also identified ventilation (89%), wearing gloves (62 and 60%) and masks (44 and 46%). In Table 4, Q9, 61 and 60% wrongly identified handwashing after coughing or sneezing as a means to prevent spread of GI infection.
- In the RT scenario (Table 5, Q12) both CG and IG groups inappropriately identified disinfecting the toilet (81 and 82%) and wearing gloves (41 and 44%) as effective interventions. Low intention to wear masks may reflect belief that social disadvantages outweighed health benefits.

#### 3.2.2 . Misperceptions about germs, dirt, cleanliness and hygiene

The extent to which participants selected inappropriate options for Q1,2,3 and 6 about hygiene comprehension and Targeted Hygiene suggests that participants from both groups believe that untargeted cleaning is an important hygiene action. These findings concur with data generated by public polling in UK (15, 16) and across 25 European countries (17) during 2019–2022 which indicated similar misunderstanding of hygiene.

However, looking at individual questions in Table 2, the extent to which the IG opted for incorrect or inappropriate responses was significantly lower compared with the CG indicating the TH video intervention increased comprehension of the difference between Targeted Hygiene and cleanliness:

- Q1 and Q2 showed that 41% of the CG and 29% of the IG believed harmful germs come into the home “via dirty floors and surfaces.” Additionally, 77% of the CG and 60% of the IG believed “harmful germs breed on dirty surfaces and spread on their own.”
- Q3 showed that only 22% of the CG and 34% of the IG agreed that the time to practise hygiene is “at key moments when there is risk of harmful germs spreading.” By contrast 64% of the CG compared with 52% of the IG said you should practise hygiene “as often as you can.”
- Q6 showed that only 33% of the CG compared with 45% of the IG selected Targeted Hygiene as the “best way to reduce risk of spread of harmful microbes.” By contrast, 36% of the CG but only 31% of the IG selected “Keeping our home clean and washing hands frequently,” and 31% of the CG compared with 24% of the IG selected “deep cleaning with antibacterial products as often as possible.”

By contrast,

- Q11and 12 (Table 5) suggest that in real-life scenarios, the TH video had no significant impact on misperceptions about cleanliness. Both groups (61 and 58% for RT scenario and 69 and 70% for the GI scenario) expressed intention to carry out non-targeted, environmental cleaning. This suggests that, although the TH video helped build confidence in Targeted Hygiene, in real-life risk scenarios participants felt they needed added protection from “deep cleaning.”

## 4 Discussion

### 4.1 Communicating targeted hygiene through storytelling

The initial aim of this study was to carry out a controlled trial to quantify whether communicating Targeted Hygiene through storytelling can improve hygiene comprehension and intention to adopt targeted hygiene behaviour. The trial showed that viewing the TH video increased hygiene comprehension, particularly of why targeting practices is an effective approach. Importantly, improved comprehension was mainly about the *journey of the germ* and targeting of practices (Q1-4). Q5-6 showed both groups could identify key hygiene moments (using the toilet, after handling raw food, before eating ready-to-eat foods, laundering, etc).

When asked about behavioural intentions in real-life risk scenarios, both groups demonstrated comprehension that handwashing reduces pathogen spread during food handling (Q7) and infection scenarios (Q9 and10), and the need to combine handwashing with actions at critical control points, such as contact surfaces, to halt *the journey of the germ* (Q8,11,12).

However, both groups appeared unable to single out the correct practices for the particular scenario, i.e., they were equally able to identify “key hygiene habits”, but both had difficulties in differentiating vital actions, from actions that would have little benefit in that scenario. This aligns with a 2021 IFH poll (15) showing that, as well as identifying correct moments for hand hygiene, people also selected hygiene actions unlikely to reduce the spread of COVID-19. It suggests improved comprehension does not necessarily translate into reasoned targeted behaviour; instead, individuals tended to resort to ingrained habits most likely shaped by family advice, school education, and public messaging.

Although not the initial aim of the study, the results support previous findings (15–17) highlighting strong public beliefs that non-targeted home cleanliness is important for preventing infection. In Q2, 77% of the CG believed harmful microbes multiply freely and move across living environments. Despite the TH video clarifying this as a misconception, 60% of the IG still selected this option. It is arguable that, people who believe pathogens are widely and randomly distributed, are unlikely to believe Targeted Hygiene optimises infection risk reduction. Consistent with this (Table 5), 69% (CG) and 70% (IG) in the GI infection, and 61% (CG) and 58% (IG) in the RT infection scenarios, expressed intention to “deep clean” in addition to performing targeted behaviours.

Whilst encouraging non-targeted domestic hygiene, rather than focusing on selected key moments in risk scenarios, may appear to have little consequence, there are important drawbacks. A non-targeted approach may undermine development of resilient behaviour by making it harder, particularly in outbreak situations or when someone is at increased risk and needs to shield, for individuals to recognise and prioritise the actions that provide maximum protection over those with limited benefit. In addition, non-targeted hygiene is resource-intensive in terms of cost, chemicals, time and effort which could ultimately reduce motivation to maintain effective hygiene practices. For these reasons Targeted Hygiene is a more sustainable approach. Whilst this trial was confined to the UK, the 2020 European poll (17) revealed limited comprehension of hygiene and cleanliness across all 25 European countries. Despite cultural differences, data from individual countries were remarkably consistent, indicating this is an issue throughout the region.

The trial findings align with the COM-B behaviour change model (18) and its application to hygiene behaviour change (19, 20). It suggests that, prior to intervention, both the CG and IG had “hygiene capability” – they knew the key moments for practising hygiene. Capability was also increased by viewing the TH video, because it increased comprehension of the benefits of Targeted Hygiene. What both groups appeared to lack was an essential component of the COM-B model which is appropriate “hygiene motivation.” Whilst comprehension may have motivated intentions of some participants to practise Targeted Hygiene, for others it may have motivated non-targeted cleaning, triggered by pre-existing beliefs and general fear of germs, which may have acted as a barrier to effective behaviour (21, 22).

### 4.2 Advancing strategies to overcome barriers to targeted hygiene behaviour

Although watching the TH video failed to increase intention to practise Targeted Hygiene in real-life scenarios, it revealed two important findings. Firstly, participants already had fairly good recall of key hygiene moments. Secondly, engaging the public through an intervention which visualized *the journey of the germ*, could have the potential both to increase hygiene comprehension, and reduce misconceptions about cleanliness. Insights from the study suggest need for further research to understand how to transform learned habits into reasoned, situation-appropriate hygiene behaviours and explore the disconnect between comprehension and intention to select appropriate hygiene habits for specific scenarios. Equally importantly this must be combined with measures to overcome barriers to effective behaviour due to poor hygiene understanding, and persistent misbeliefs about germs.

Our data suggest people place considerable importance on visual cleanliness and have an instinctive desire to avoid environments perceived to be dirty (23, 24). Emphasis on visible cleanliness may conflict with public health recommendations that promote Targeted Hygiene rather than routine, non-targeted cleaning, potentially leading to confusion about appropriate behaviours. Consequently, affirming the mental and physical benefits of cleanliness, whilst clearly positioning it within the framework of Targeted Hygiene, is an important component of hygiene communication.

Health agencies increasingly recognise the need to capitalise on sustained improvement in hygiene knowledge observed since the COVID-19 pandemic (25). If we can find a way to build on the public’s existing recall of key hygiene habits, as identified in this study, this offers a cost-effective strategy for promoting hygiene behaviour change.

## 5 Limitations of the study

An obvious limitation of this study is that it involved hypothetical online scenarios, rather than measuring actual behaviour change. However, taking the findings from earlier polling (15–17) we reasoned that, before doing human observation work, we need to learn more about the disconnect between hygiene comprehension and intended behaviour, and gain deeper insights into how and why the public misunderstand hygiene concepts, and how this might be addressed. It was concluded that this was best achieved through this online desk trial.

We are also concerned that providing participants with self-reported, multiple-choice options, as in all our polling and the trial, may have influenced their responses and reduced the need for independent reasoning. Although the overall increase in comprehension recorded by the trial was modest, it was achieved through a short-term exposure to the principles of Targeted Hygiene (the 7-min TH video was viewed only once). For certain questions the increase in comprehension was up to 20% in the IG compared with the CG. The impact of longer-term exposure to Targeted Hygiene messaging requires investigation. To build on the findings of this trial, we have started further studies using focus groups to obtain more in-depth understanding of hygiene comprehension, behavioural intentions and barriers to change, and the impact of interventions promoting Targeted Hygiene.

## 6 Conclusion

Our findings suggest that people understand the need for hygiene practices but lack understanding of when and where to apply them. The study suggests that animated visual materials, now increasingly accessible and affordable, could, in future, be used to enhance hygiene comprehension and dispel misperceptions that hinder change, thereby motivating development of effective and time-efficient hygiene behaviour.

Hygiene resilience is essential for tackling AMR (26), future epidemics and pandemics, and improving community healthcare (27). It also allows government agencies to weigh the benefits of advising or mandating enhanced or additional hygiene measures, against the costs of disrupting social, work and educational activities, and later relaxing them, without losing public confidence and engagement.

## Data Availability

The raw data supporting the conclusions of this article will be made available by the authors, without undue reservation.

## Footnotes

1
https://www.bi.team/

2
https://www.bi.team/bi-ventures/predictiv/
